# Effects of osteopathic treatment on pulmonary function and chronic thoracic pain after coronary artery bypass graft surgery (OstinCaRe): study protocol for a randomised controlled trial

**DOI:** 10.1186/s12906-016-1468-3

**Published:** 2016-11-25

**Authors:** Gert Roncada

**Affiliations:** 1 Jessa Hospital, Heart Centre Hasselt, Stadsomvaart 11, 3500 Hasselt, Belgium; 2 Commission for Osteopathic Research, Practice and Promotion, Mechelen, Belgium

**Keywords:** Osteopathic treatment, Coronary artery bypass graft surgery, Slow vital capacity, Pulmonary function, Chronic thoracic pain

## Abstract

**Background:**

Coronary artery bypass graft surgery (CABG) is an effective and widespread coronary revascularisation technique, nevertheless there are a number of long-term postoperative complications from which patients can suffer. One year after CABG surgery pulmonary function is decreased by 12% and 30% of the patients suffer from chronic thoracic pain. To date and to our knowledge there are no effective treatments for these conditions. The aim of the present clinical trial is to explore the effectiveness of osteopathic treatment on these conditions.

**Methods:**

The study is designed as a randomised controlled trial with two parallel groups. Group A will receive a standard cardiac rehabilitation programme during 12 weeks and group B will receive the same standard cardiac rehabilitation programme supplemented with four osteopathic treatments (OT). OT will be performed at week 4, 5, 8 and 12 after surgery. Three hundred and eight patients (Group A: *n* = 154, Group B: *n* = 154) will be enrolled from the cardiothoracic surgery department of the Jessa Hospital Hasselt. Blinding will be assured for the staff of the cardiac rehabilitation centre and outcome assessors. Primary outcome measure will be the mean difference in change from baseline in slow vital capacity (SVC) at 12 weeks after surgery between groups. Secondary outcome measures will be the change from baseline in quality of life, pain, thoracic stiffness and maximal aerobic capacity at 12 weeks after surgery. A follow-up is planned 52 weeks after surgery for SVC, quality of life, pain and thoracic stiffness. Intention to treat analysis will be executed.

**Discussion:**

The OstinCare study has been designed to explore the potential long-term added value of osteopathic treatment in the management of decreased pulmonary function, chronic thoracic pain and diminished thoracic mobility after CABG surgery.

**Trial registration:**

The protocol has been retrospectively registered on ClinicalTrials.gov (NCT01714791).

## Background

Approximately 640,000 coronary artery bypass graft (CABG) surgery procedures are performed in Europe and the United States each year to restore or optimize myocardial perfusion in coronary artery disease [1, 2]. Worldwide there are approximately over two million open-heart surgeries per year [3]. In this surgical intervention, venous and/or arterial grafts are used to bypass the coronary occlusion or stenosis. Because cardioplegia (induction of temporary cardiac arrest) is routinely used, the patient’s circulation is coupled to a cardiopulmonary bypass and access to the heart is most often achieved by median sternotomy. After CABG surgery, a hospital stay of 1 to 2 weeks is generally required [4, 5].

Although CABG surgery is an effective coronary revascularisation technique, there are a number of postoperative complications from which patients can suffer. For example, a decrease in pulmonary function is a frequently observed complication after CABG surgery. During the first week after CABG surgery vital capacity (VC) decreases by 30–60% [6–9] and even up to 1 year this remains reduced by 12% [10, 11]. Reduced VC has a negative effect on exercise tolerance (Vo2max) [12] and therefore it is important to optimize pulmonary function after CABG surgery. No method of postoperative therapy has been distinguished in treating or preventing these long-term changes [6].

In addition, Ragnarsdòttir et al. [8] found a diminished mobility of the left hemithorax at 3 months after CABG surgery. This decreased thoracic mobility was still present 12 months after surgery [11]. Thoracic mobility and vital capacity were affected more when the left internal thoracic artery (LITA)-retractor was used and reduced thoracic mobility is related to a diminished pulmonary function [10].

Chronic pain, which is defined as pain without apparent biological value that has persisted beyond the normal tissue healing time, which usually takes 3 months [13] after CABG surgery. Chronic pain after CABG surgery is described in several studies reaching a time period from 3 to 28 months [14–20]. Kehlet et al. [14] reported a pain prevalence of 30–50%, from which 5–10% suffer from severe disabling pain after more than 6 months after surgery. Numerous other studies report a pain prevalence of 28–56% from 3 to 28 months after CABG surgery [15–20]. Many theories for its cause have been proposed in literature, but the aetiology is still not clear and no therapy or technique has been shown to reduce chronic pain after CABG surgery [17, 21]. In literature this syndrome is described as chronic chest pain [19], chronic thoracic pain [18], chronic post-sternotomy pain [14–20].

Chronic pain after CABG surgery is a major clinical problem, which is distressing and reduces the quality of life of patients [14].

As a result, many patients undergoing CABG surgery suffer from decreased pulmonary function, reduced thoracic mobility and/or chronic thoracic pain. These anomalies have significant clinical repercussion and may have an effect on the patients’ quality of life [14].

According to current clinical guidelines, exercise intervention should be initiated early after CABG surgery [22]. According to these recommendations, exercise training should be individually tailored according to the clinical condition, baseline exercise capacity and ventricular function. Upper-body training can begin when the sternal wound is stable. The general applicable exercise training in cardiac rehabilitation consists of walking, jogging, cycling, swimming, rowing, stair climbing, elliptical trainers and aerobic dancing, at low to moderate exercise intensity, for 3–5 days/week. Programmes should last up to 12 weeks for outpatient settings. However in this trajectory, pulmonary function, thoracic pain and thoracic mobility are not specifically targeted.

In fact, to our knowledge there are no effective treatments to treat the latter conditions or effective preventive interventions. Osteopathic treatment (OT) has been used to treat and manage pain symptoms. Several articles have been published addressing acute and chronic pain in different medical conditions [23–25]. However, no trials have been conducted to test the effect of OT on chronic pain after CABG surgery.

## Methods/Design

### Aim of the study

The aim of this randomised controlled trial is to examine whether OT could lead to a better treatment of chronic thoracic pain, decreased pulmonary function and/or decreased thoracic mobility.

We hypothesized that OT reduces the decrease in SVC, reduces chronic thoracic pain, reduces thoracic stiffness and improves the quality of life in patients at 12 weeks and 52 weeks after CABG surgery.

### Design

OstinCaRe stands for Osteopathy in Cardiac Rehabilitation. The OstinCare-study is designed as a randomised controlled trial with two parallel groups. Group A will receive a standard exercise-based cardiac rehabilitation programme and group B will receive a standard exercise-based cardiac rehabilitation programme with four additional osteopathic treatments. The study will be performed at the Jessa Hospital Hasselt, Belgium.

### Participants

Subjects admitted to the hospital for elective CABG with median sternotomy will be eligible for this study. Participant recruitment began in January 2010 and is expected to finish in December 2017. Subjects with diagnosed chronic obstructive pulmonary disease, diagnosed neurologic disease that prevents participation in the cardiac rehabilitation programme, diagnosed nephrological disease that requires haemodialysis, prior thoracic surgery, surgery in the epigastric, right or left hypochondriac region will be excluded. Subjects will also be excluded if the subject has a prolonged stay (>5 days) in the intensive care unit. All CABG surgery procedures will be performed by the same surgical team. Subjects may not receive any other manual treatment on the spine and/or thorax during the study.

### Randomisation and masking

**Fig. 1:**
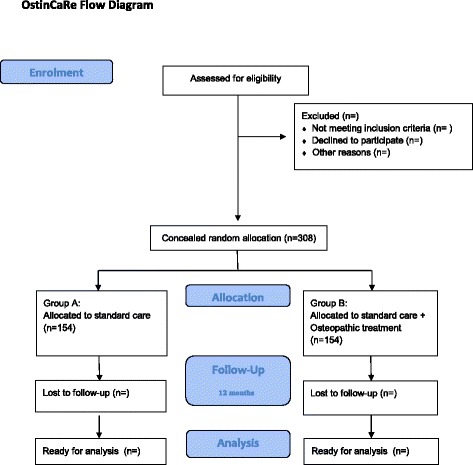
Flow of participants during the conduct of the trial

**Fig. 2:**
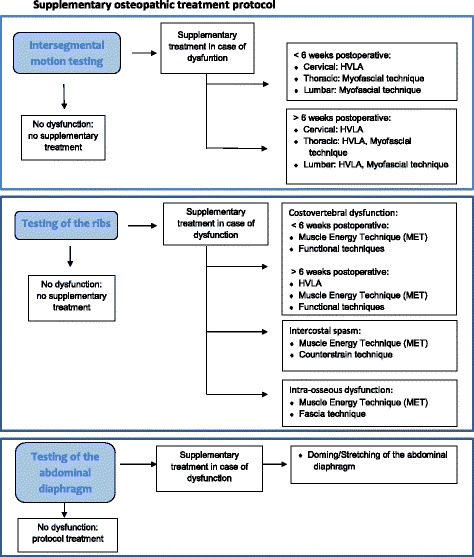
Study content for the schedule of enrolment, interventions, and assessments. -t_2_: preoperative, -t_1_: 9^th^ postoperative day, t_0_: start of cardiac rehabilitation programme (3 weeks postoperative), t_1_: 4 weeks postoperative, t_2_: 5 weeks postoperative, t_3_: 9 weeks postoperative, t_4_: 12 weeks postoperative, t_5_: 12 months postoperative. SVC: slow vital capacity. VAS: visual analogue scale. MacNew QLQ: MacNew quality of life questionnaire

Patients will be randomly assigned in a 1:1 ratio to either group A or group B (Fig. 1). A blocked allocation schedule will be used. Randomisation will be performed by means of opaque, sealed envelopes. A physical therapist of the cardiac rehabilitation centre will perform and store the randomisation. The personnel of the cardiac rehabilitation centre performing the outcome measurements in this study are unaware of patient’s allocation. Osteopathic treatments will not be performed in the presence of the personnel of the cardiac rehabilitation centre. The treatments will be performed in another location to assure that the personnel of the cardiac rehabilitation centre remains blinded to patient’s allocation. Only the treating osteopaths will be aware of the patient’s allocation. The enrolment and procedures are visualised in Fig. 2 according to the Standard Protocol Items: Recommendations for Interventional Trials (SPIRIT) guidelines [26, 27].

### Intervention

#### Cardiac rehabilitation programme

The cardiac rehabilitation programme is a multidisciplinary programme in line with the current guidelines. Components of the multidisciplinary programme include patient assessment, physical activity counselling, exercise training, diet/nutritional counselling, weight control management, lipid management, blood pressure monitoring, smoking cessation, and psychosocial management [22].

The outpatient exercise-based cardiac rehabilitation programme includes endurance training. No strength training exercises are executed. According to cardiac rehabilitation literature, all patients exercise under close supervision 3 days per week for a total duration of 3 months [28], and because this frequency is easily attainable for most patients. Exercise training intensity is determined by baseline Vo2peak assessment [29]. Patients exercise at a heart rate corresponding to 65% of baseline Vo2peak. Each exercise training session takes 45 min. Exercise time is apportioned as follows: 42% on the cycle ergometer, 33% on the treadmill and 25% on the arm-cranking device [30].

#### Osteopathic procedure

The protocol used for OT and osteopathic examination (OE) incorporates a number of osteopathic techniques and will be performed by five registered osteopaths with a minimum experience of 5 years. The protocol used is based on the work of Dickey [31] and supplemented with the findings of other authors [32–35]. The most common findings found in literature are:

- Decrease in costal mobility [8, 10, 11, 36]
- Decrease in pulmonary function [6, 8, 10]
- Decreased thoracic mobility [8, 10, 11, 31]
- Dysfunction of the abdominal diaphragm [31, 37–41]

The nomenclature, indications and contraindications for the OE and OT are based on the work of Nicholas and Nicholas [32], Chila [42], on the professional competence profile of an osteopath [43–45] and the benchmarks of the World Health Organisation [46].

The OE protocol is a set of standardised test for a first evaluation of the patient. The findings are noted on a predefined Microsoft excel-file by the Osteopath.

- Inspection: in standing, seated and supine: observe the patient in posterior, anterior and lateral view to develop the most complete understanding of the patients makeup before performing the remainder of the OE ([32] p. 3–14).
- Position test of the thoracic spine (seated) ([42] p.561)
- Intersegmental motion testing:
  - thoracic seated ([32] p. 42–49)
  - cervical supine ([32] p. 60–65)
  - costal supine ([32] p. 52–59
- Costal motion testing supine [32] (p.53–56)
- Evaluation of the abdominal diaphragm (seated and supine) ([42] p. 567)

**Fig. 3:**
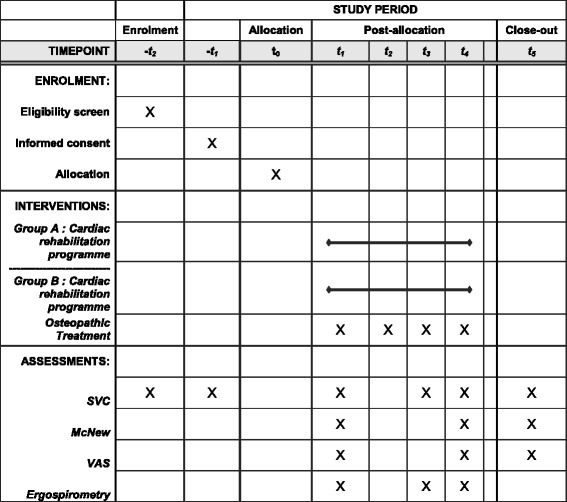
Supplementary osteopathic treatment protocol. Abbreviations: HVLA: high velocity low amplitude manipulation; MET: muscle energy technique

The OT consists of a standardised treatment protocol (Table 1) and a supplementary treatment protocol of the dysfunctions found during the examination. In order to be reproducible, the different treatment possibilities are discussed and presented (Fig. 3). The OT will take 30–45 min. The examination room is air-conditioned and has a constant temperature of 21–22 °C. The OT will be performed at 4 weeks postoperative (t_1_), 5 weeks postoperative (t_2_), 9 weeks postoperative (t_3_) and at 12 weeks postoperative (t_4_). OT can be considered as safe, and major adverse events are very rare [47, 48]. In case adverse events should occur, they will be recorded and discussed in the final paper.

**Table 1:** Standard treatment protocol OstinCaRe study

Osteopathic technique	Description	Rationale for use
Doming/stretching of the abdominal diaphragm [31–33]	Direct release of the respiratory diaphragm: the patient is supine and the osteopath stands on the homolateral side of the patient. The osteopath places the cubital side of the heterolateral hand under the anterior costal margin and the fingers of the homolateral hand under the posterior costal margin. During inspiration, the hands follow the expansion of the ribs and during expiration, the osteopath holds the expansion of the ribs. This is repeated 3–4 times on each side.	Improves motion of diaphragm Releases connective tissue tension within structures of the thorax
Myofascial release of the thorax [33]	One hand is placed posterior on one hemi thorax, the other hand anterior of the same hemi thorax (according to the anatomy of the ribs). Determine the direction of free movement with passive motion testing. Maintain either indirect or direct position until release. The osteopath stimulates the expression of the fasciae after the release.	Releases tissue restriction Promotes improved lymphatic and venous drainage Improves pulmonary function and lymphatic circulation
Suboccipital inhibition [31, 33, 34]	Fingertips are placed on occipital condyles. The osteopath applies an outward and cephalad traction to decompress the occipital joint	Improves parasympathetic function Releases restricted tissues around vagus nerves
Equilibration anterior-posterior [35]	One hand is placed under the sacrum and one hand on the sternum. The osteopath follows and synchronizes the expression of the primary respiration between sacrum and sternum. The same is done between the occiput and the sternum.	Improves lymphatic and venous circulation

### Outcome measures

Primary outcome measure will be the mean difference in change from baseline in SVC at 12 weeks after surgery between the two groups.

Secondary outcome measures will include:

- Change from baseline in SVC at 52 weeks
- Change from baseline in MacNew QLQ at 12 and 52 weeks after surgery
- Change in pain from baseline on Visual Analogue Scale (VAS) at 12 and 52 weeks after surgery
- Change in thoracic stiffness from baseline on VAS at 12 and 52 weeks after surgery
- Change from baseline in maximal aerobic capacity (VO2max) at 12 weeks after surgery

#### Spirometry

The Pocket-Spiro USB100 (Medical Electronic Construction & Logistic nv, Belgium) will be used for measuring pulmonary function. All patients are asked to perform a slow vital capacity (SVC) test, consisting of three measurements. For VC the best of the three measurements is used and for IVC the average of three measurements is used [49]. The instrument is calibrated prior to the test. When an OT is planned, SVC will be measured at least 2 days after OT, because the study wants to measure long-term effects (effects of the entire intervention), instead of any short-term effect (within the first hours after OT). SVC will be measured preoperative (-t_2_), 9^th^ postoperative day (-t_1_), 4 weeks after surgery (t_1_), 8 weeks after surgery (t_3_), 12 weeks after surgery (t_4_) and at 12 months after surgery (t_5_).

#### Ergospirometry

All patients will perform a maximal cardiopulmonary exercise test on a cycle ergometer [50]. All the exercise tests will be performed at the same time of day (between 8.30 and 11.30 am). The test will be performed at 4 weeks after surgery (t_1_), 9 weeks after surgery (t_3_) and at 12 weeks after surgery (t_4_).

During the exercise test, an electronically braked e-Bike (Acertys) is used. The cycling frequency is set at 70 cycles/min. In addition, exercise tests will be prematurely ended when myocardial ischemia and/or severe ventricular arrhythmias would occur and the subject will be excluded from the study. Both the starting and incremental cycling resistance will be set between 10 and 40 W and increased every minute to volitional fatigue.

Pulmonary gas exchange analysis will be performed by using cardiopulmonary ergospirometry device (Jaeger MasterScreen CPX). Before every test, a gas and volume calibration will be executed. During the test, environmental temperature is kept stable (19–21 °C). Oxygen uptake, expiratory volume and respiratory exchange ratio are collected breath-by-breath and averaged every 10 s. Using a 12-lead ECG device, heart rate is monitored and averaged every 10 s. In addition, maximal cycling resistance and total test duration are reported. By V-slope method, ventilatory threshold is calculated. The criteria for defining a maximal cardiopulmonary exercise test are an achieved heart rate > 85% of the maximal predicted heart rate, and/or a Respiratory gas Exchange Ratio (RER) > 1,09 [51].

#### MacNew quality of life questionnaire (QLQ) and Visual analogue scale (VAS)

The Flemish version of the MacNew QLQ and the VAS for pain and thoracic stiffness are delivered to the patients by a blinded person of the cardiac rehabilitation centre at enrolment (t_0_), 12 weeks after surgery (t_4_) and at 12 months after surgery (t_5_). The Flemish version of the MacNew QLQ demonstrates good psychometric properties and is recommended as a specific instrument for assessing and evaluate health-related quality of life in Flemish-speaking patients [52].

### Data management

All procedures comply with confidentiality standards for medical data. Authorized medical staff treating the patient is granted unconstrained access to the patients’ data, whereas restricted access to anonymized data is granted to other local staff and researchers. All data will be entered electronically and all original forms will be kept at the study site. Participant files are stored in alphabetical order and stored in a secure and accessible place and manner at the study site. Participant files will be maintained in storage for a period of 3 years after completion of the study. During this time, access to the completely encrypted dataset can be obtained on individual demand. A back-up of all data will be performed every week on a USB-stick and on the hospitals’ backup server. Because the known minimal risk of this study a data monitoring committee is not needed [53]. Important protocol modifications during this study will be communicated to the trial registry and the journal of publication.

### Sample size

A priori sample size calculation is based on pulmonary function (by GPower 3.1). The hypothesis for the OstinCaRe study is that the addition of OT towards endurance exercise training increases inspiratory vital capacity (IVC) by 12% during the follow-up of CABG patients. Power analysis is based on a 12% decrease in IVC at 12 weeks after surgery [11]. As a result, an increase of approximately 12% of the IVC is expected at 12 weeks after CABG. Sample size is computed considering an effect size of 0.50, a statistical power of 0.80 and an alpha level of 0.05. The power analysis outcome defines that 128 subjects per group are needed. Based on unpublished data of the cardiac rehabilitation centre a dropout rate of 20% is to be expected. Therefore, 154 subjects per group are needed. Participant recruitment began in January 2010 and is expected to finish in December 2017. Interim analysis is planned when 154 (50%) patients are included. The study can be stopped before reaching sample size if there is a significant change in surgery technique, which could compromise reproducibility and comparability throughout the study. The author makes the final decision to terminate the trial.

### Statistical analysis

Data will be analysed by a statistician blinded to group allocations, using the Statistical Package for the Social Sciences (SPSS) v. 22.0 (IBM). First, descriptive statistics will be executed, with calculation of means and standard deviations, and analysis of data distribution (by Shapiro-Wilk test) and evaluation of outliers. In case of normal data distribution, one-way ANOVA with repeated measures will be executed to analyse and compare changes in parameters between groups (with Bonferroni corrections for multiple comparisons). Relations between parameters will be examined by Pearson correlations. In case of non-normal data distribution, absolute changes in parameters will be compared between groups by Mann Whitney U-tests (with Bonferroni corrections for multiple comparisons). Relations between parameters will be examined by Spearman correlations. Statistical significance is set at *p* < 0.05, two-tailed. Observed statistical power will be calculated by use of GPower v. 3.1. Intention to treat analysis will be executed. Missing data will be handled using the last observation carried forward imputation technique. Dropouts and withdrawals from the study will be recorded through the intervention and follow-up periods. When differences in baseline phenotype are present, these differences will be taken into account during analysis of treatment effect between groups, by regarding them as co-variates.

## Discussion

Although CABG surgery is an effective coronary revascularisation technique, there are a number of postoperative complications, such as diminished pulmonary function and chronic thoracic pain, from which patients are prone to suffer from [10, 11, 14–20]. To our knowledge, there are no effective treatments to treat these conditions or effective preventive interventions [6, 17, 21]. The OstinCaRe study has been designed to explore the potential long-term added value of OT in the management of decreased pulmonary function, chronic thoracic pain and diminished thoracic mobility after CABG surgery. The present study is the first study to examine long-term effects of OT after CABG surgery using rigorous procedures and gold standard methods for clinical trials. Previous studies studied the short-term effects of OT after CABG surgery. One study has proven that OT has immediate, beneficial haemodynamic effects after CABG surgery when administered while the patient is sedated [34]. Another study mentioned beneficial, though statistically insignificant, effect of OT on length of stay and recovery of bowel function of CABG surgical patients [54].

The expected outcomes from the present study will be increased pulmonary function, reduction in thoracic pain and increased thoracic mobility. The study has the potential to deliver the first valuable complement in cardiac rehabilitation programmes to address these problems.

## Abbreviations

CABG: Coronary artery bypass graft
HVLA: High velocity low amplitude
IVC: Inspiratory vital capacity
LITA: Left internal thoracic artery
MET: Muscle energy technique
OE: Osteopathic examination
OT: Osteopathic treatment
QLQ: Quality of life questionnaire
RER: Respiratory gas exchange ratio
SVC: Slow vital capacity
VAS: Visual analogue scale
VC: Vital capacity
